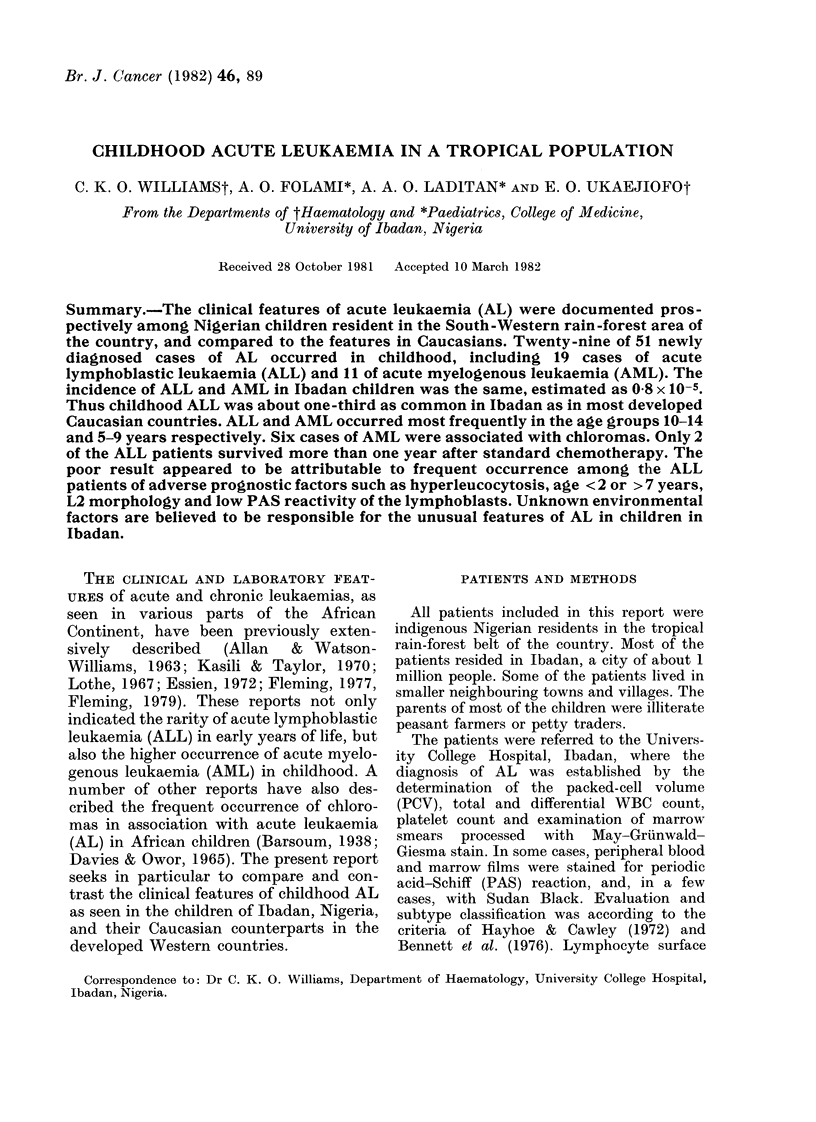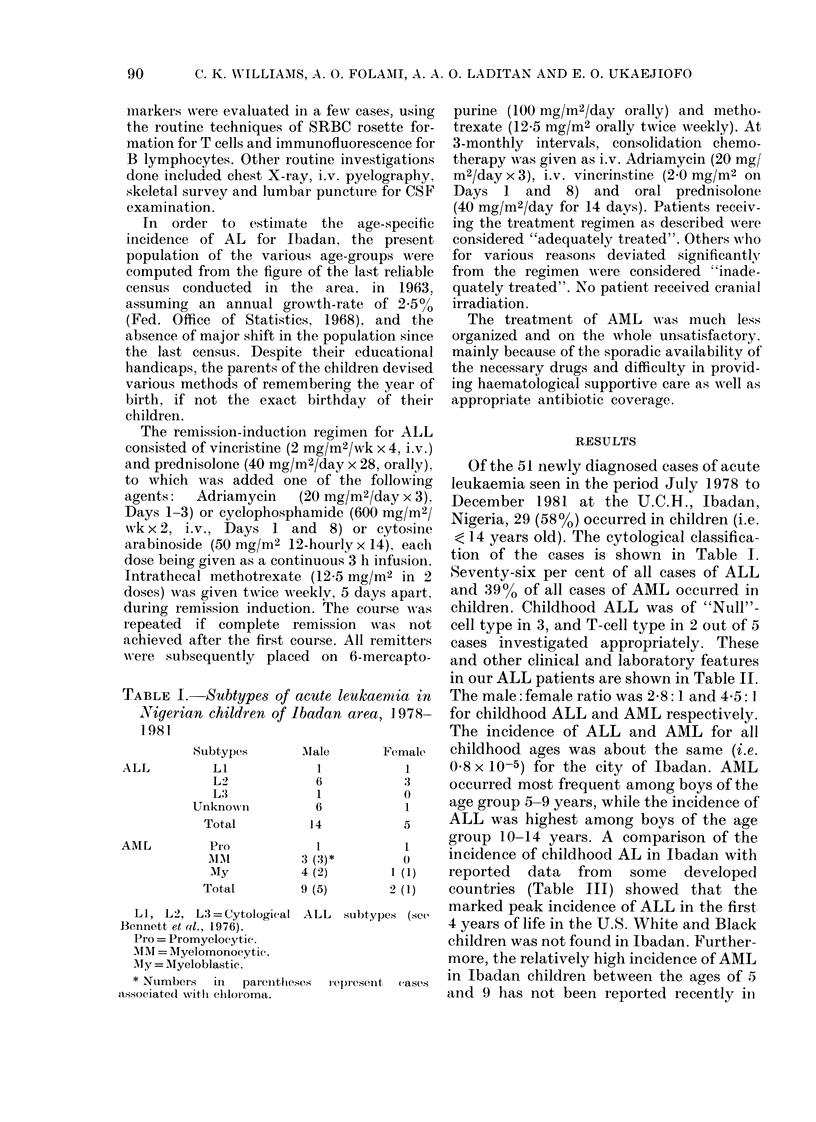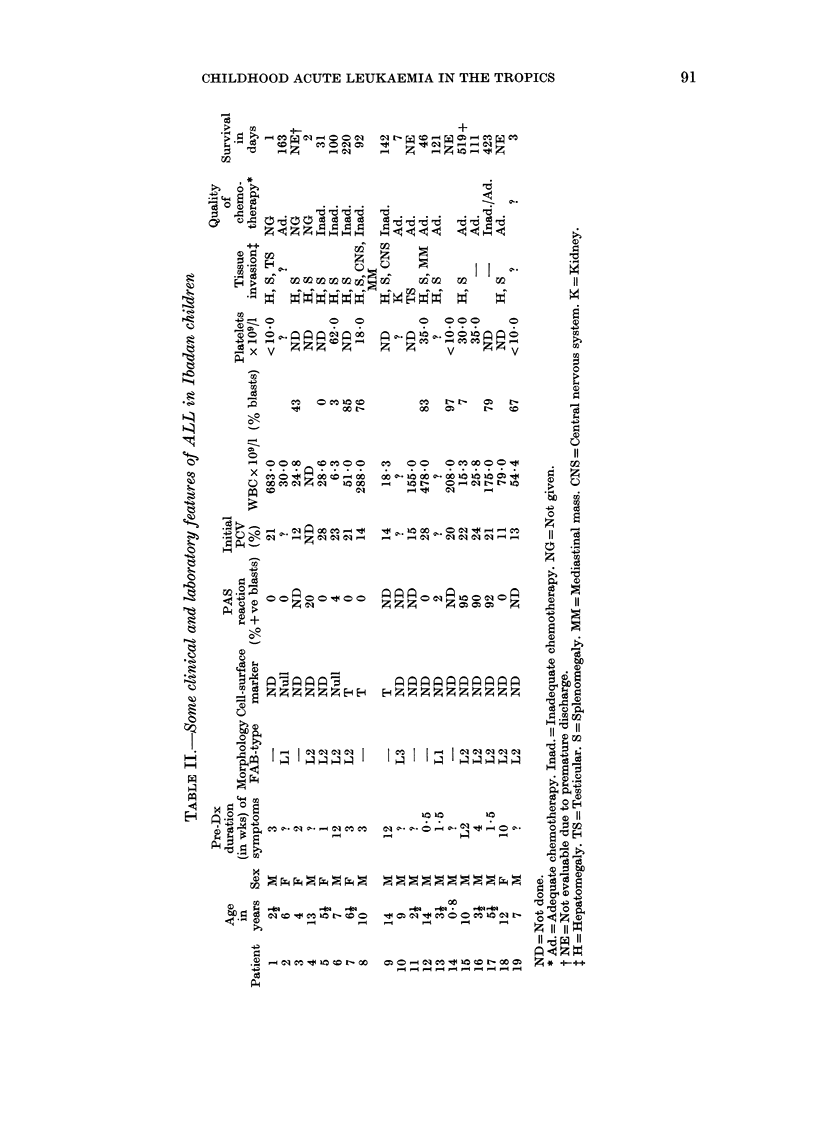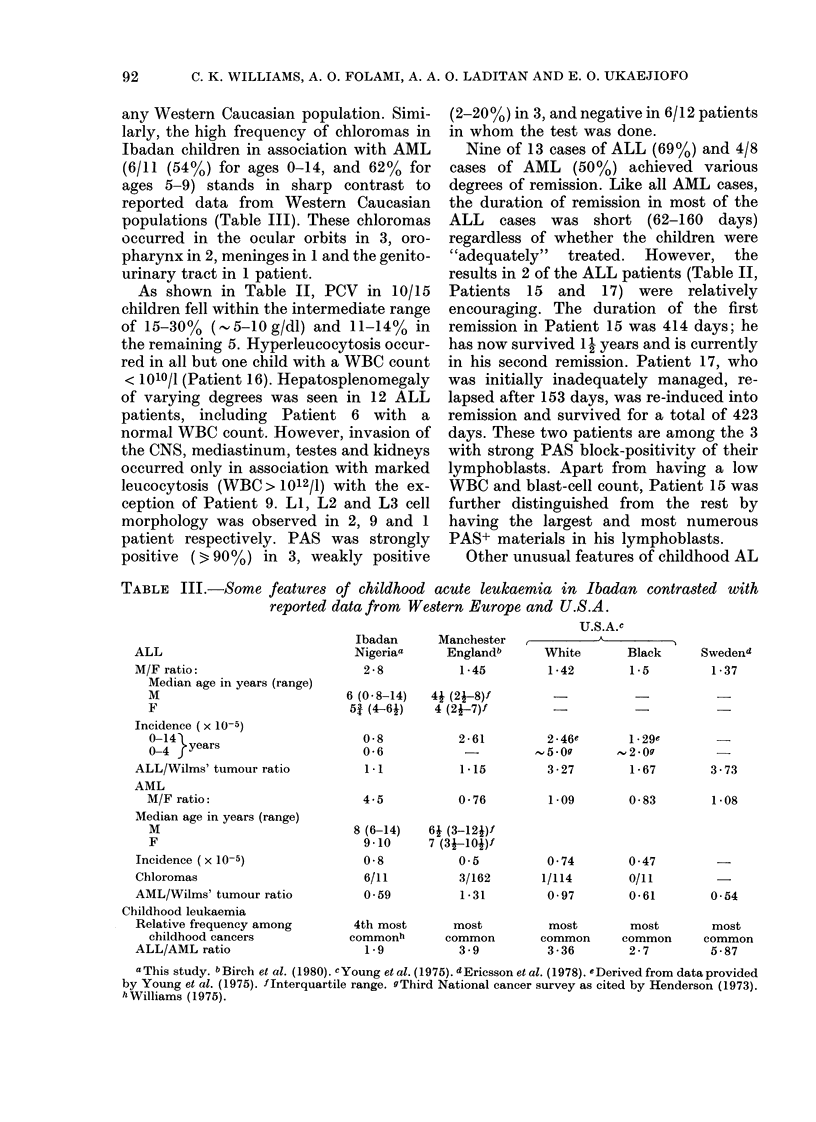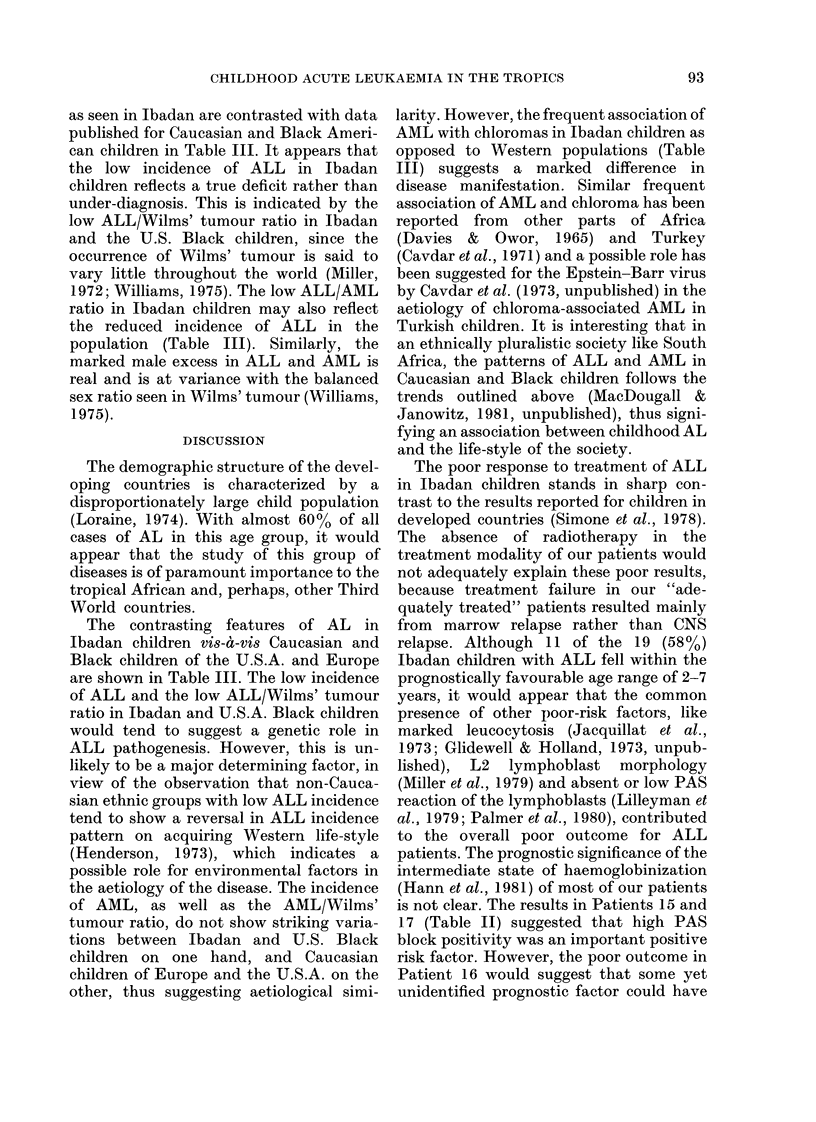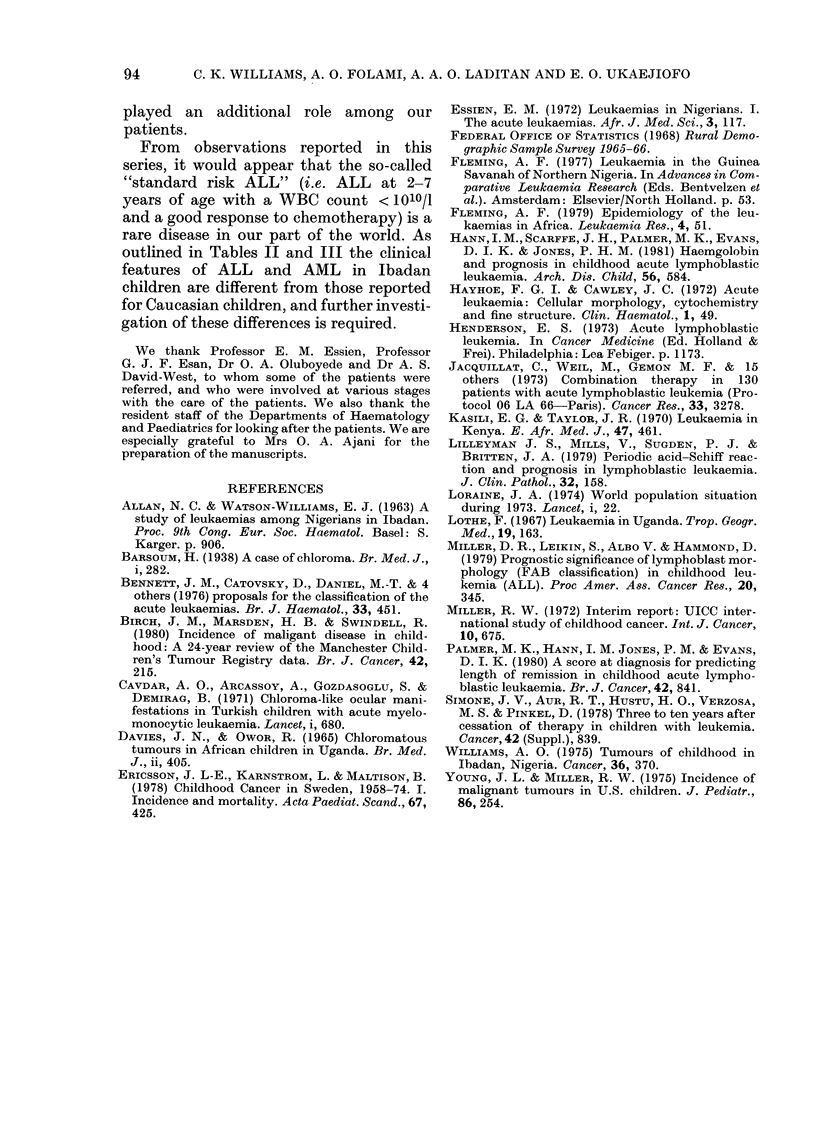# Childhood acute leukaemia in a tropical population.

**DOI:** 10.1038/bjc.1982.169

**Published:** 1982-07

**Authors:** C. K. Williams, A. O. Folami, A. A. Laditan, E. O. Ukaejiofo

## Abstract

The clinical features of acute leukaemia (AL) were documented prospectively among Nigerian children resident in the South-Western rain-forest area of the country, and compared to the features in Caucasians. Twenty-nine of 51 newly diagnosed cases of AL occurred in childhood, including 19 cases of acute lymphoblastic leukaemia (ALL) and 11 of acute myelogenous leukaemia (AML). The incidence of ALL the AML in Ibadan children was the same, estimated as 0.8 X 10(-5). Thus childhood ALL was about one-third as common in Ibadan as in most developed Caucasian countries. ALL and AML occurred most frequently in the age groups 10-14 and 5-9 years respectively. Six cases of AML were associated with chloromas. Only 2 of the ALL patients survived more than one year after standard chemotherapy. The poor result appeared to be attributable to frequent occurrence among the ALL patients of adverse prognostic factors such as hyperleucocytosis, age less than 2 or greater than 7 years, L2 morphology and low PAS reactivity of the lymphoblasts. Unknown environmental factors are believed to be responsible for the unusual features of AL in children in Ibadan.


*Br. J. Cancer* (1982) **46**, 89

### CHILDHOOD ACUTE LEUKAEMIA IN A TROPICAL POPULATION

C. K. O. WILLIAMS†, A. O. FOLAMI*, A. A. O. LADITAN* AND E. O. UKAEJIOFO†

*From the Departments of †Haematology and *Paediatrics, College of Medicine, University of Ibadan, Nigeria*

Received 28 October 1981 Accepted 10 March 1982

**Summary.—The clinical features of acute leukaemia (AL) were documented prospectively among Nigerian children resident in the South-Western rain-forest area of the country, and compared to the features in Caucasians. Twenty-nine of 51 newly diagnosed cases of AL occurred in childhood, including 19 cases of acute lymphoblastic leukaemia (ALL) and 11 of acute myelogenous leukaemia (AML). The incidence of ALL and AML in Ibadan children was the same, estimated as $0{\cdot}8 \times 10^{-5}$. Thus childhood ALL was about one-third as common in Ibadan as in most developed Caucasian countries. ALL and AML occurred most frequently in the age groups 10–14 and 5–9 years respectively. Six cases of AML were associated with chloromas. Only 2 of the ALL patients survived more than one year after standard chemotherapy. The poor result appeared to be attributable to frequent occurrence among the ALL patients of adverse prognostic factors such as hyperleucocytosis, age <2 or >7 years, L2 morphology and low PAS reactivity of the lymphoblasts. Unknown environmental factors are believed to be responsible for the unusual features of AL in children in Ibadan.**

THE CLINICAL AND LABORATORY FEATURES of acute and chronic leukaemias, as seen in various parts of the African Continent, have been previously extensively described (Allan & Watson-Williams, 1963; Kasili & Taylor, 1970; Lothe, 1967; Essien, 1972; Fleming, 1977, Fleming, 1979). These reports not only indicated the rarity of acute lymphoblastic leukaemia (ALL) in early years of life, but also the higher occurrence of acute myelogenous leukaemia (AML) in childhood. A number of other reports have also described the frequent occurrence of chloromas in association with acute leukaemia (AL) in African children (Barsoum, 1938; Davies & Owor, 1965). The present report seeks in particular to compare and contrast the clinical features of childhood AL as seen in the children of Ibadan, Nigeria, and their Caucasian counterparts in the developed Western countries.

### PATIENTS AND METHODS

All patients included in this report were indigenous Nigerian residents in the tropical rain-forest belt of the country. Most of the patients resided in Ibadan, a city of about 1 million people. Some of the patients lived in smaller neighbouring towns and villages. The parents of most of the children were illiterate peasant farmers or petty traders.

The patients were referred to the University College Hospital, Ibadan, where the diagnosis of AL was established by the determination of the packed-cell volume (PCV), total and differential WBC count, platelet count and examination of marrow smears processed with May–Grünwald–Giesma stain. In some cases, peripheral blood and marrow films were stained for periodic acid–Schiff (PAS) reaction, and, in a few cases, with Sudan Black. Evaluation and subtype classification was according to the criteria of Hayhoe & Cawley (1972) and Bennett *et al.* (1976). Lymphocyte surface

Correspondence to: Dr C. K. O. Williams, Department of Haematology, University College Hospital, Ibadan, Nigeria.

markers were evaluated in a few cases, using the routine techniques of SRBC rosette formation for T cells and immunofluorescence for B lymphocytes. Other routine investigations done included chest X-ray, i.v. pyelography, skeletal survey and lumbar puncture for CSF examination.

In order to estimate the age-specific incidence of AL for Ibadan, the present population of the various age-groups were computed from the figure of the last reliable census conducted in the area, in 1963, assuming an annual growth-rate of 2·5% (Fed. Office of Statistics, 1968), and the absence of major shift in the population since the last census. Despite their educational handicaps, the parents of the children devised various methods of remembering the year of birth, if not the exact birthday of their children.

The remission-induction regimen for ALL consisted of vincristine (2 mg/m$^2$/wk × 4, i.v.) and prednisolone (40 mg/m$^2$/day × 28, orally), to which was added one of the following agents: Adriamycin (20 mg/m$^2$/day × 3), Days 1–3) or cyclophosphamide (600 mg/m$^2$/wk × 2, i.v., Days 1 and 8) or cytosine arabinoside (50 mg/m$^2$ 12-hourly × 14), each dose being given as a continuous 3 h infusion. Intrathecal methotrexate (12·5 mg/m$^2$ in 2 doses) was given twice weekly, 5 days apart, during remission induction. The course was repeated if complete remission was not achieved after the first course. All remitters were subsequently placed on 6-mercaptopurine (100 mg/m$^2$/day orally) and methotrexate (12·5 mg/m$^2$ orally twice weekly). At 3-monthly intervals, consolidation chemotherapy was given as i.v. Adriamycin (20 mg/m$^2$/day × 3), i.v. vincristine (2·0 mg/m$^2$ on Days 1 and 8) and oral prednisolone (40 mg/m$^2$/day for 14 days). Patients receiving the treatment regimen as described were considered "adequately treated". Others who for various reasons deviated significantly from the regimen were considered "inadequately treated". No patient received cranial irradiation.

The treatment of AML was much less organized and on the whole unsatisfactory, mainly because of the sporadic availability of the necessary drugs and difficulty in providing haematological supportive care as well as appropriate antibiotic coverage.

TABLE I.—*Subtypes of acute leukaemia in Nigerian children of Ibadan area*, 1978–1981

| | Subtypes | Male | Female |
|---|---|---|---|
| ALL | L1 | 1 | 1 |
| | L2 | 6 | 3 |
| | L3 | 1 | 0 |
| | Unknown | 6 | 1 |
| | Total | 14 | 5 |
| AML | Pro | 1 | 1 |
| | MM | 3 (3)* | 0 |
| | My | 4 (2) | 1 (1) |
| | Total | 9 (5) | 2 (1) |

L1, L2, L3 = Cytological ALL subtypes (see Bennett *et al.*, 1976).

Pro = Promyelocytic.

MM = Myelomonocytic.

My = Myeloblastic.

* Numbers in parentheses represent cases associated with chloroma.

### RESULTS

Of the 51 newly diagnosed cases of acute leukaemia seen in the period July 1978 to December 1981 at the U.C.H., Ibadan, Nigeria, 29 (58%) occurred in children (i.e. ⩽ 14 years old). The cytological classification of the cases is shown in Table I. Seventy-six per cent of all cases of ALL and 39% of all cases of AML occurred in children. Childhood ALL was of "Null"-cell type in 3, and T-cell type in 2 out of 5 cases investigated appropriately. These and other clinical and laboratory features in our ALL patients are shown in Table II. The male:female ratio was 2·8:1 and 4·5:1 for childhood ALL and AML respectively. The incidence of ALL and AML for all childhood ages was about the same (*i.e.* $0{\cdot}8 \times 10^{-5}$) for the city of Ibadan. AML occurred most frequent among boys of the age group 5–9 years, while the incidence of ALL was highest among boys of the age group 10–14 years. A comparison of the incidence of childhood AL in Ibadan with reported data from some developed countries (Table III) showed that the marked peak incidence of ALL in the first 4 years of life in the U.S. White and Black children was not found in Ibadan. Furthermore, the relatively high incidence of AML in Ibadan children between the ages of 5 and 9 has not been reported recently in

TABLE II.—*Some clinical and laboratory features of ALL in Ibadan children*

| Patient | Age in years | Sex | Pre-Dx duration (in wks) of symptoms | Morphology FAB-type | Cell-surface marker | PAS reaction (% + ve blasts) | Initial PCV (%) | WBC × $10^9$/l | (% blasts) | Platelets × $10^9$/l | Tissue invasion‡ | Quality of chemo-therapy* | Survival in days |
|---|---|---|---|---|---|---|---|---|---|---|---|---|---|
| 1 | 2½ | M | 3 | — | ND | 0 | 21 | 683·0 | | < 10·0 | H, S, TS | NG | 1 |
| 2 | 6 | F | ? | L1 | Null | 0 | ? | 30·0 | | ? | ? | Ad. | 163 |
| 3 | 4 | F | 2 | — | ND | ND | 12 | 24·8 | 43 | ND | H, S | NG | NE† |
| 4 | 13 | M | ? | L2 | ND | 20 | ND | ND | | ND | H, S | NG | 2 |
| 5 | 5½ | F | 1 | L2 | ND | 0 | 28 | 28·6 | 0 | ND | H, S | Inad. | 31 |
| 6 | 7 | M | 12 | L2 | Null | 4 | 23 | 6·3 | 3 | 62·0 | H, S | Inad. | 100 |
| 7 | 6½ | F | 3 | L2 | T | 0 | 21 | 51·0 | 85 | ND | H, S | Inad. | 220 |
| 8 | 10 | M | 3 | — | T | 0 | 14 | 288·0 | 76 | 18·0 | H, S, CNS, MM | Inad. | 92 |
| 9 | 14 | M | 12 | — | T | ND | 14 | 18·3 | | ND | H, S, CNS | Inad. | 142 |
| 10 | 9 | M | ? | L3 | ND | ND | ? | ? | | ? | K | Ad. | 7 |
| 11 | 2½ | M | ? | — | ND | ND | 15 | 155·0 | | ND | TS | Ad. | NE |
| 12 | 14 | M | 0·5 | — | ND | 0 | 28 | 478·0 | 83 | 35·0 | H, S, MM | Ad. | 46 |
| 13 | 3½ | M | 1·5 | L1 | ND | 2 | ? | ? | | ? | H, S | Ad. | 121 |
| 14 | 0·8 | M | ? | — | ND | ND | 20 | 208·0 | 97 | < 10·0 | | | NE |
| 15 | 10 | M | L2 | L2 | ND | 95 | 22 | 15·3 | 7 | 30·0 | H, S | Ad. | 519+ |
| 16 | 3½ | M | 4 | L2 | ND | 90 | 24 | 25·8 | | 35·0 | — | Ad. | 111 |
| 17 | 5½ | M | 1·5 | L2 | ND | 92 | 21 | 175·0 | 79 | ND | — | Inad./Ad. | 423 |
| 18 | 12 | F | 10 | L2 | ND | 0 | 11 | 79·0 | | ND | H, S | Ad. | NE |
| 19 | 7 | M | ? | L2 | ND | ND | 13 | 54·4 | 67 | < 10·0 | ? | ? | 3 |

ND = Not done.

* Ad. = Adequate chemotherapy. Inad. = Inadequate chemotherapy. NG = Not given.

† NE = Not evaluable due to premature discharge.

‡ H = Hepatomegaly. TS = Testicular. S = Splenomegaly. MM = Mediastinal mass. CNS = Central nervous system. K = Kidney.

any Western Caucasian population. Similarly, the high frequency of chloromas in Ibadan children in association with AML (6/11 (54%) for ages 0–14, and 62% for ages 5–9) stands in sharp contrast to reported data from Western Caucasian populations (Table III). These chloromas occurred in the ocular orbits in 3, oropharynx in 2, meninges in 1 and the genitourinary tract in 1 patient.

As shown in Table II, PCV in 10/15 children fell within the intermediate range of 15–30% (~5–10 g/dl) and 11–14% in the remaining 5. Hyperleucocytosis occurred in all but one child with a WBC count $< 10^{10}$/l (Patient 16). Hepatosplenomegaly of varying degrees was seen in 12 ALL patients, including Patient 6 with a normal WBC count. However, invasion of the CNS, mediastinum, testes and kidneys occurred only in association with marked leucocytosis (WBC $> 10^{12}$/l) with the exception of Patient 9. L1, L2 and L3 cell morphology was observed in 2, 9 and 1 patient respectively. PAS was strongly positive ($\geqslant 90\%$) in 3, weakly positive (2–20%) in 3, and negative in 6/12 patients in whom the test was done.

Nine of 13 cases of ALL (69%) and 4/8 cases of AML (50%) achieved various degrees of remission. Like all AML cases, the duration of remission in most of the ALL cases was short (62–160 days) regardless of whether the children were "adequately" treated. However, the results in 2 of the ALL patients (Table II, Patients 15 and 17) were relatively encouraging. The duration of the first remission in Patient 15 was 414 days; he has now survived 1½ years and is currently in his second remission. Patient 17, who was initially inadequately managed, relapsed after 153 days, was re-induced into remission and survived for a total of 423 days. These two patients are among the 3 with strong PAS block-positivity of their lymphoblasts. Apart from having a low WBC and blast-cell count, Patient 15 was further distinguished from the rest by having the largest and most numerous PAS+ materials in his lymphoblasts.

Other unusual features of childhood AL

TABLE III.—*Some features of childhood acute leukaemia in Ibadan contrasted with reported data from Western Europe and U.S.A.*

| ALL | Ibadan Nigeria[a] | Manchester England[b] | U.S.A.[c] White | U.S.A.[c] Black | Sweden[d] |
|---|---|---|---|---|---|
| M/F ratio: | 2·8 | 1·45 | 1·42 | 1·5 | 1·37 |
| Median age in years (range) | | | | | |
| M | 6 (0·8–14) | 4½ (2½–8)[f] | — | — | — |
| F | 5¾ (4–6½) | 4 (2½–7)[f] | — | — | — |
| Incidence ($\times 10^{-5}$) | | | | | |
| 0–14 years | 0·8 | 2·61 | 2·46[e] | 1·29[e] | — |
| 0–4 years | 0·6 | — | ~5·0[g] | ~2·0[g] | — |
| ALL/Wilms' tumour ratio | 1·1 | 1·15 | 3·27 | 1·67 | 3·73 |
| AML | | | | | |
| M/F ratio: | 4·5 | 0·76 | 1·09 | 0·83 | 1·08 |
| Median age in years (range) | | | | | |
| M | 8 (6–14) | 6½ (3–12½)[f] | | | |
| F | 9·10 | 7 (3½–10½)[f] | | | |
| Incidence ($\times 10^{-5}$) | 0·8 | 0·5 | 0·74 | 0·47 | — |
| Chloromas | 6/11 | 3/162 | 1/114 | 0/11 | — |
| AML/Wilms' tumour ratio | 0·59 | 1·31 | 0·97 | 0·61 | 0·54 |
| Childhood leukaemia | | | | | |
| Relative frequency among childhood cancers | 4th most common[h] | most common | most common | most common | most common |
| ALL/AML ratio | 1·9 | 3·9 | 3·36 | 2·7 | 5·87 |

[a]This study. [b]Birch *et al.* (1980). [c]Young *et al.* (1975). [d]Ericsson *et al.* (1978). [e]Derived from data provided by Young *et al.* (1975). [f]Interquartile range. [g]Third National cancer survey as cited by Henderson (1973). [h]Williams (1975).

as seen in Ibadan are contrasted with data published for Caucasian and Black American children in Table III. It appears that the low incidence of ALL in Ibadan children reflects a true deficit rather than under-diagnosis. This is indicated by the low ALL/Wilms' tumour ratio in Ibadan and the U.S. Black children, since the occurrence of Wilms' tumour is said to vary little throughout the world (Miller, 1972; Williams, 1975). The low ALL/AML ratio in Ibadan children may also reflect the reduced incidence of ALL in the population (Table III). Similarly, the marked male excess in ALL and AML is real and is at variance with the balanced sex ratio seen in Wilms' tumour (Williams, 1975).

### DISCUSSION

The demographic structure of the developing countries is characterized by a disproportionately large child population (Loraine, 1974). With almost 60% of all cases of AL in this age group, it would appear that the study of this group of diseases is of paramount importance to the tropical African and, perhaps, other Third World countries.

The contrasting features of AL in Ibadan children *vis-à-vis* Caucasian and Black children of the U.S.A. and Europe are shown in Table III. The low incidence of ALL and the low ALL/Wilms' tumour ratio in Ibadan and U.S.A. Black children would tend to suggest a genetic role in ALL pathogenesis. However, this is unlikely to be a major determining factor, in view of the observation that non-Caucasian ethnic groups with low ALL incidence tend to show a reversal in ALL incidence pattern on acquiring Western life-style (Henderson, 1973), which indicates a possible role for environmental factors in the aetiology of the disease. The incidence of AML, as well as the AML/Wilms' tumour ratio, do not show striking variations between Ibadan and U.S. Black children on one hand, and Caucasian children of Europe and the U.S.A. on the other, thus suggesting aetiological similarity. However, the frequent association of AML with chloromas in Ibadan children as opposed to Western populations (Table III) suggests a marked difference in disease manifestation. Similar frequent association of AML and chloroma has been reported from other parts of Africa (Davies & Owor, 1965) and Turkey (Cavdar *et al.*, 1971) and a possible role has been suggested for the Epstein–Barr virus by Cavdar *et al.* (1973, unpublished) in the aetiology of chloroma-associated AML in Turkish children. It is interesting that in an ethnically pluralistic society like South Africa, the patterns of ALL and AML in Caucasian and Black children follows the trends outlined above (MacDougall & Janowitz, 1981, unpublished), thus signifying an association between childhood AL and the life-style of the society.

The poor response to treatment of ALL in Ibadan children stands in sharp contrast to the results reported for children in developed countries (Simone *et al.*, 1978). The absence of radiotherapy in the treatment modality of our patients would not adequately explain these poor results, because treatment failure in our "adequately treated" patients resulted mainly from marrow relapse rather than CNS relapse. Although 11 of the 19 (58%) Ibadan children with ALL fell within the prognostically favourable age range of 2–7 years, it would appear that the common presence of other poor-risk factors, like marked leucocytosis (Jacquillat *et al.*, 1973; Glidewell & Holland, 1973, unpublished), L2 lymphoblast morphology (Miller *et al.*, 1979) and absent or low PAS reaction of the lymphoblasts (Lilleyman *et al.*, 1979; Palmer *et al.*, 1980), contributed to the overall poor outcome for ALL patients. The prognostic significance of the intermediate state of haemoglobinization (Hann *et al.*, 1981) of most of our patients is not clear. The results in Patients 15 and 17 (Table II) suggested that high PAS block positivity was an important positive risk factor. However, the poor outcome in Patient 16 would suggest that some yet unidentified prognostic factor could have played an additional role among our patients.

From observations reported in this series, it would appear that the so-called "standard risk ALL" (*i.e.* ALL at 2–7 years of age with a WBC count $<10^{10}/l$ and a good response to chemotherapy) is a rare disease in our part of the world. As outlined in Tables II and III the clinical features of ALL and AML in Ibadan children are different from those reported for Caucasian children, and further investigation of these differences is required.

We thank Professor E. M. Essien, Professor G. J. F. Esan, Dr O. A. Oluboyede and Dr A. S. David-West, to whom some of the patients were referred, and who were involved at various stages with the care of the patients. We also thank the resident staff of the Departments of Haematology and Paediatrics for looking after the patients. We are especially grateful to Mrs O. A. Ajani for the preparation of the manuscripts.

### REFERENCES

ALLAN, N. C. & WATSON-WILLIAMS, E. J. (1963) A study of leukaemias among Nigerians in Ibadan. *Proc. 9th Cong. Eur. Soc. Haematol.* Basel: S. Karger. p. 906.

BARSOUM, H. (1938) A case of chloroma. *Br. Med. J.*, i, 282.

BENNETT, J. M., CATOVSKY, D., DANIEL, M.-T. & 4 others (1976) proposals for the classification of the acute leukaemias. *Br. J. Haematol.*, **33**, 451.

BIRCH, J. M., MARSDEN, H. B. & SWINDELL, R. (1980) Incidence of maligant disease in childhood: A 24-year review of the Manchester Children's Tumour Registry data. *Br. J. Cancer*, **42**, 215.

CAVDAR, A. O., ARCASSOY, A., GOZDASOGLU, S. & DEMIRAG, B. (1971) Chloroma-like ocular manifestations in Turkish children with acute myelomonocytic leukaemia. *Lancet*, i, 680.

DAVIES, J. N., & OWOR, R. (1965) Chloromatous tumours in African children in Uganda. *Br. Med. J.*, ii, 405.

ERICSSON, J. L-E., KARNSTROM, L. & MALTISON, B. (1978) Childhood Cancer in Sweden, 1958–74. I. Incidence and mortality. *Acta Paediat. Scand.*, **67**, 425.

ESSIEN, E. M. (1972) Leukaemias in Nigerians. I. The acute leukaemias. *Afr. J. Med. Sci.*, **3**, 117.

FEDERAL OFFICE OF STATISTICS (1968) *Rural Demographic Sample Survey 1965–66.*

FLEMING, A. F. (1977) Leukaemia in the Guinea Savanah of Northern Nigeria. In *Advances in Comparative Leukaemia Research* (Eds. Bentvelzen *et al.*). Amsterdam: Elsevier/North Holland. p. 53.

FLEMING, A. F. (1979) Epidemiology of the leukaemias in Africa. *Leukaemia Res.*, **4**, 51.

HANN, I. M., SCARFFE, J. H., PALMER, M. K., EVANS, D. I. K. & JONES, P. H. M. (1981) Haemgolobin and prognosis in childhood acute lymphoblastic leukaemia. *Arch. Dis. Child*, **56**, 584.

HAYHOE, F. G. I. & CAWLEY, J. C. (1972) Acute leukaemia: Cellular morphology, cytochemistry and fine structure. *Clin. Haematol.*, **1**, 49.

HENDERSON, E. S. (1973) Acute lymphoblastic leukemia. In *Cancer Medicine* (Ed. Holland & Frei). Philadelphia: Lea Febiger. p. 1173.

JACQUILLAT, C., WEIL, M., GEMON M. F. & 15 others (1973) Combination therapy in 130 patients with acute lymphoblastic leukemia (Protocol 06 LA 66—Paris). *Cancer Res.*, **33**, 3278.

KASILI, E. G. & TAYLOR, J. R. (1970) Leukaemia in Kenya. *E. Afr. Med. J.*, **47**, 461.

LILLEYMAN J. S., MILLS, V., SUGDEN, P. J. & BRITTEN, J. A. (1979) Periodic acid–Schiff reaction and prognosis in lymphoblastic leukaemia. *J. Clin. Pathol.*, **32**, 158.

LORAINE, J. A. (1974) World population situation during 1973. *Lancet*, i, 22.

LOTHE, F. (1967) Leukaemia in Uganda. *Trop. Geogr. Med.*, **19**, 163.

MILLER, D. R., LEIKIN, S., ALBO V. & HAMMOND, D. (1979) Prognostic significance of lymphoblast morphology (FAB classification) in childhood leukemia (ALL). *Proc Amer. Ass. Cancer Res.*, **20**, 345.

MILLER, R. W. (1972) Interim report: UICC international study of childhood cancer. *Int. J. Cancer*, **10**, 675.

PALMER, M. K., HANN, I. M. JONES, P. M. & EVANS, D. I. K. (1980) A score at diagnosis for predicting length of remission in childhood acute lymphoblastic leukaemia. *Br. J. Cancer*, **42**, 841.

SIMONE, J. V., AUR, R. T., HUSTU, H. O., VERZOSA, M. S. & PINKEL, D. (1978) Three to ten years after cessation of therapy in children with leukemia. *Cancer*, **42** (Suppl.), 839.

WILLIAMS, A. O. (1975) Tumours of childhood in Ibadan, Nigeria. *Cancer*, **36**, 370.

YOUNG, J. L. & MILLER, R. W. (1975) Incidence of malignant tumours in U.S. children. *J. Pediatr.*, **86**, 254.